# Effect of the Spatial Distribution of the Temperature and Humidity Index in a New Zealand White Rabbit House on Respiratory Frequency and Ear Surface Temperature

**DOI:** 10.3390/ani11061657

**Published:** 2021-06-02

**Authors:** Maria Alice Junqueira Gouvêa Silva, Patrícia Ferreira Ponciano Ferraz, Luana Mendes dos Santos, Gabriel Araújo e Silva Ferraz, Giuseppe Rossi, Matteo Barbari

**Affiliations:** 1 Department of Animal Science, Federal University of Lavras (UFLA), Lavras 37200-900, Minas Gerais, Brazil; 2 Department of Agricultural Engineering, Federal University of Lavras (UFLA), Lavras 37200-900, Minas Gerais, Brazil; patricia.ponciano@ufla.br (P.F.P.F.); luanna_mendess@yahoo.com.br (L.M.d.S.); gabriel.ferraz@ufla.br (G.A.eS.F.); 3 Department of Agriculture, Food, Environment and Forestry, University of Firenze, 50145 Firenze, Italy; giuseppe.rossi@unifi.it (G.R.); matteo.barbari@unifi.it (M.B.)

**Keywords:** thermal environment, rabbit farming, thermal stress, geostatistics, physiological responses

## Abstract

**Simple Summary:**

A rabbit house’s microclimate has a strong influence on the physiological responses of rabbits, and thermal discomfort may damage their development. The primary aim of this study was to develop a spatial distribution of temperature and humidity index (THI) maps via kriging interpolation, as well as to characterize and evaluate its relationship with physiological responses (i.e., the respiratory frequency (RF) and ear surface temperature (EST) of New Zealand white (NZW) rabbits kept in a rabbit house. The spatial distribution maps of THI allowed us to visualize the heterogeneity in the distribution space of the variables, as well as to identify regions where NZW rabbits were exposed to unfavorable developmental conditions. The relationship between THI, RF, and EST was evident, and the maps showed that an increase in THI led to an acceleration of respiratory movements and an elevation of surface temperature in rabbit ears. This led to an attempt to dissipate excess heat acquired from the environment. Thus, the spatial distribution maps of THI overlaid with RF and EST data was found to provide useful information to assist the producer in making decisions to improve the production environment for NZW rabbits.

**Abstract:**

The objective of this study was to characterize and evaluate the temperature and humidity index (THI) of New Zealand white (NZW) rabbits kept in a rabbit house using geostatistical techniques. Furthermore, we sought to evaluate its relationship with respiratory frequency (RF) and ear surface temperature (EST). The experiment was conducted at the Federal University of Lavras, Brazil. A total of 52 NZW rabbits were used. For the characterization of the thermal environment, the dry bulb temperature (t_db_, °C), relative humidity (RH, %), and dew point temperature (t_dp_, °C) were collected at 48 points in the rabbit house at 6:00 a.m., 12:00 p.m., and 6:00 p.m. for seven days. The RF and EST of the animals was monitored. Subsequently, the THI was calculated and the data were analyzed using geostatistical tools and kriging interpolation. In addition, the RF and EST data were superimposed on the rabbit house’s THI data maps. The magnitude of the variability and structure of the THI inside the rabbit house were characterized and the heterogeneity was visualized. Critical THI points inside the rabbit house and in locations where animals with high RF and ESTs were housed were identified, thus providing information about improving the production environment.

## 1. Introduction

Consumers today are more aware of the relationship between diet and health, which has increased the demand for healthier foods [1]. Thus, the consumption of rabbit meat has increase due to its high dietary value, its lipid component, its low fat and cholesterol content, its high unsaturated fatty acid content, and its good n–6/n–3 polyunsaturated fatty acid ratio. Therefore, rabbit meat has a positive impact on consumer health [1,2,3]. As a result, modern society is increasingly aware of welfare issues in rabbit farming systems [4].

Rabbits are highly sensitive to high air temperatures (t_air_, °C) and relative humidity (RH, %) conditions (t_air_ > 26 °C and RH > 70%) because they are covered in fur and have few sweat glands, which limits their ability to dissipate excess body heat [5]. According to [6], high t_air_ can have harmful effects on the growth and reproductive performance of rabbits. This represents a problem in rabbit farming in hot climate zones and in Mediterranean countries [7].

A number of physiological processes, including respiratory frequency (RF, breaths.min^−1^) and ear surface temperature (EST, °C), are related to the adaptive responses used by animals to minimize the impact of a stressor [8,9]. According to [10,11], when an animal’s body temperature increases, the RF increases, which is an adaptive response to dissipate excess heat by evaporation and homeothermy maintenance. According to these authors, this change is one of the first symptoms presented by rabbits when they experience thermal discomfort.

EST is considered another important thermoregulation mechanism for rabbits due to the presence of thermal receptors present in their ears [12]. According to [13], the ears of rabbits are composed of a vasomotor mechanism in which it controls the blood circulation from the body’s nucleus to the blood vessels and capillaries, which can be dilated and contracted by a vasomotor tool that favors the dissipation of superficial heat from the ear.

Therefore, the microclimate of a rabbit house has a strong influence on rabbit farming by affecting the physiological responses of the animals. This characterizes the environmental conditions of these animals within the production system, which is of paramount importance [11]. In this context, the development of new technologies that improve existing housing environments at a low production cost is necessary to better understand the effects of thermal conditions on rabbit farming and to assist producers in obtaining maximum productivity [10,14].

Environmental variables, such as dry bulb temperature (t_db_, °C) and RH, as well as climatic indices, such as the temperature and humidity index (THI), can be evaluated inside a facility through spatialization and geostatistics [14,15]. The use of geostatistics makes it possible to quantitatively characterize the spatial variability of these variables inside a rabbit house environment, preventing biased interpretations of data and allowing the observation of spatial dependence through kriging maps [13,14,15,16]. 

The geostatistical techniques are considered an efficient tool for evaluating thermal variables inside of animal facilities. Such techniques have been used by many researchers studying the thermal comfort of other animal species such as broilers, dairy cows, laying hens, and free-range broilers [17,18,19,20,21].

In tropical countries such as Brazil, only a few studies have evaluated the thermal comfort of rabbit production facilities; thus, farmers and researchers have searched for computational tools that can assist in the evaluation and decision-making processes for supporting better animal production environments [14].

According to [14,22], geostatistical mapping using kriging maps can be a highly important tool for analyzing the environmental and physiological conditions of animals in production systems because these maps are easily interpreted.

Therefore, the objective of this study was to characterize and evaluate the THI and its relationship with the RF and EST of rabbits kept in a rabbit house with a ridge vent near the spatial distribution of the THI. This was done using isometric maps generated by kriging interpolation.

## 2. Materials and Methods

### 2.1. Housing and Animals

This experiment was conducted in a rabbit house in the Cuniculture sector of the Department of Animal Science, Federal University of Lavras (Universidade Federal de Lavras, UFLA), Minas Gerais, Brazil. We used 52 New Zealand white (NZW) rabbits (28 males and 24 females) that were 58 days old. The rabbits were randomly housed in 26 collective galvanized wire cages (2 animals per cage). During the experimental period, the animals had free access to balanced feed and drinking water.

**Figure 1:**
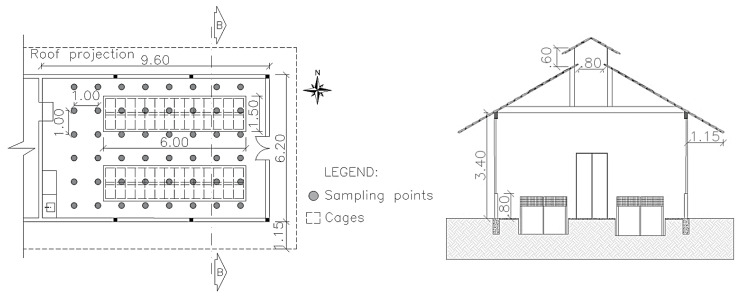
Rabbit house scheme. (**Left**) Floor plan of the rabbit house and position of sampling points. (**Right**) Cut BB of the rabbit house section. Source: adapted from [11,14].

The rabbit house studied was oriented in an east–west direction and measured 6.20 m in width, 9.60 m in length, and 3.0 m in ceiling height, with a 1.0 m roof overhang (Figure 1). The roof was built with clay tiles with a 30° slope. The floor was concrete, with two 1.50 × 6.00 m and 0.80 m deep cement collecting ditches at the center of the house. The side walls were made of masonry measuring 0.80 m in height.

The rabbit house had a ridge vent to aid in the air renewal and ventilation of the internal environment. The ridge vent opening over the roof tile was measured as 5% of the total width of the shed (0.30 m) and had a slope of 20% of the total width of the shed (1.70 m). The opening was covered with wire mesh to prevent birds from flying in.

### 2.2. Environmental and Physiological Response Measurements

In May 2016, the t_db_ (°C), RH (%), RF (breaths.min^−1^), and EST (°C) data were collected at three different times of the day for seven days: 6:00 a.m., 12:00 p.m., and 6:00 p.m.

The RF was assessed by counting the flank movements of the rabbits using a digital chronometer (± 0.01 s). The respiratory movements of the animals were monitored for 15 s and then multiplied by four to obtain the breaths per minute (breaths.min^−1^) [23].

The EST (°C) was measured at three different points on the animal’s ear (base, middle, and tip), and the mean of the values was calculated. An infrared thermometer (Raytek Raynger ST, Raytek Corporation, accuracy of ±0.1 °C) was used with the emissivity control set to 0.95 [11].

To characterize the thermal environment inside the rabbit house, measurements of the t_db_ (°C), dew point temperature (t_dp_, °C), and RH (%) were taken for 1 min at a height of 1 m (from the ground) at the center of the cage. To measure these variables, a digital thermohygrometer with ±0.1 °C and 1.0% accuracy, respectively (ITWH-1200, Instrutemp^®^), was used as the base station. Inside the rabbit house, the sensors were positioned in a sampling grid of 1 × 1 m (Figure 1), totaling 48 data sampling points (6 columns × 8 rows).

The collected t_db_, RH, and t_dp_ data were converted into the THI, according to the equation of [24] (Equation (1)):

(1) $$\mathrm{THI}=t_{\mathrm{db}}+0.36\times \left(t_{\mathrm{dp}}\right)+41.2$$

### 2.3. Dataset

A database containing the raw data for THI (°C), RF (breaths.min^−1^), and EST (°C) was generated for NZW rabbits. Subsequently, the spatial dependence of the THI data calculated for the rabbit house were analyzed by classic semivariogram fitting, following what was described in [14].

### 2.4. Data Partition Method for Validation of the Models

The cross-validation (CV) technique was used to fit the best semivariogram method for the studied data. According to [25], CV is a technique used to evaluate estimation errors that compares predicted and sampled values. The sampled value at a certain location, Z(si), is temporarily discarded from the dataset, and then a prediction is made by kriging at the location using the remaining samples.

Thus, some values that are useful for choosing the method—i.e., the mean error (ME), standard deviation of the mean error (SDME), reduced mean error (RE), and the standard deviation of the reduced mean error (SDRE)—can be obtained. The selection criteria based on CV can find the ME and RE values closest to zero, the lowest SDME value, and the SDRE value closest to one, according to the method of [26].

Based on the CV, the restricted maximum likelihood (REML) method was fitted. This method generally results in less biased estimates for small samples [14,27].

The qualitatively best fitting model was the wave model, which is described by [28] (Equation (2)):

(2) $$\hat{\gamma }(h)={C_{0}}^{2}+\frac{a}{h}\;\sin{\left(\frac{h}{a}\right)}^{4}$$

where h is the distance between the samples, C_0_ is the nugget effect, and a is the interval.

The fitted model differs from what has been reported in the relevant literature, specifically, vis-à-vis, research that studied environmental variables in animal facilities that was fitted to the spherical model [14,29,30,31]. The difference may be due to the sampling grid used in those studies, as well as the environmental effects and/or the experimental design. The selection of the wave model is justified because it best adapts to the fittings performed in studies on environmental variables [32].

After the semivariogram function was fitted, the data were interpolated via an ordinary kriging, which subsequently generated the spatial distribution map of the THI data.

Geostatistical analysis and the plotting of isoline maps were performed in R (R Development Core Team) using the geoR package [33]. QGIS software version 2.14.15 (Quantum GIS Development Team) was used to create the map layouts.

The RF and EST data were crossed and overlaid on to the final environmental data maps of the rabbit house.

## 3. Results and Discussion

Descriptive statistics and geostatistical techniques were used to better quantitatively and qualitatively evaluate THI, which provided important information about the area occupied by each class, as well as a way to understand their variability and influence on the studied environment. The models and parameters of the experimental semivariograms fitted for the THI in the rabbit house are shown in Table 1.

Table 1 shows the values of the nugget effect (C_0_) on the different days and times analyzed. According to [14,19], C_0_ is an important parameter of the semivariogram because it indicates unexplained variability when considering the sampling distance used. It is also possible to quantify the individual contribution of errors when the nugget effect is expressed as a percentage of the threshold (C_0_ + C_1_), thus facilitating the comparison of the degree of spatial dependence (DSD) of the variables under study [34].

When the geostatistical analyses were performed, we observed that during the experimental period, spatial variability in the THI (Table 1) appeared inside the rabbit house. Thus, the spatial distribution of the THI inside the rabbit house was not homogeneous, which can affect the physiological responses of NZW rabbits and potentially expose them to uncomfortable thermal conditions inside the facility.

The individual quantification of the nugget effect was performed according to the classification suggested by [35], where values of C_0_ < 25.00% have strong spatial dependence, C_0_ between 25.00 and 75.00% have moderate spatial dependence, and C_0_ > 75.00% have weak spatial dependence. The contribution of the variable THI throughout the experimental period showed C_0_ values below 25.00%, representing strong spatial dependence (Table 1).

The strong degree of spatial dependence of the THI found on all analyzed days and times may be related to the wave model. To illustrate this point, [18] fitted the experimental semivariograms for the variables of litter temperature (t_litter_, °C), skin surface temperature (t_skin_, °C), and relative humidity (RH, %) for chicks aged 7, 14, and 21 days using the spherical model. They found strong DSD values for RH and moderate DSD values for t_litter_ on days 7 and 14, as well as for t_skin_ on day 7. Furthermore, they noted weak DSD values for t_litter_ on day 21 and t_skin_ on days 7 and 14. The possible causes of the DSD differences found between the wave and spherical models can be attributed to the sampling grid used by the authors, as well as the environmental effects and/or the experimental design.

Between the days and times studied, the THI presented a mean error (ME) close to zero, indicating the quality of the semivariogram fit and the efficiency of the analysis, according to [36]. Moreover, [18] obtained an ME close to zero, which agreed with the findings of our study. This agreement indicated that the fits of the models are efficient in evaluating the environmental comfort variables of production animals.

Conversely, reach values (A, m) (Table 1) indicate to what extent the variable is influenced by space, and they are of great importance in determining the limit of spatial dependence [18,37]. When they were observed in this study, we found several important differences. These differences showed that the highest reach values were obtained from the fourth to the seventh day at 12 p.m. and that the lowest spatial continuity was mostly observed at 6:00 a.m. (days 1, 3, 4, 5, and 6). Thus, at 12:00 p.m., the variable showed greater spatial dependence than at 6:00 a.m., which may be related to inadequate air renewal in the environment, especially at hotter times of the day, indicating that the ridge vent was not sufficient for improving the environmental conditions inside the rabbit house.

The obtained results suggest that the index evaluated did not exhibit a random distribution in space because it had strong spatial dependencies and more than the shortest distance between the sampled points, indicating that the application of the geostatistical technique is appropriate [38]. According to [38], the occurrence of spatial dependence allows interpolation of data using the common kriging technique used to make spatial distribution maps.

Moreover, [22] suggested using geostatistical mapping via kriging maps, which could be utilized as a tool to evaluate the spatial variability of the enthalpy comfort index (ECI), litter temperature, lighting, and air velocity in broiler houses with a fogging system because these maps are easily interpreted. According to the authors, this visual tool shows areas with different spatial variability for the evaluated microclimatic attributes, helping to identify specific problematic areas inside poultry houses.

**Figure 2:**
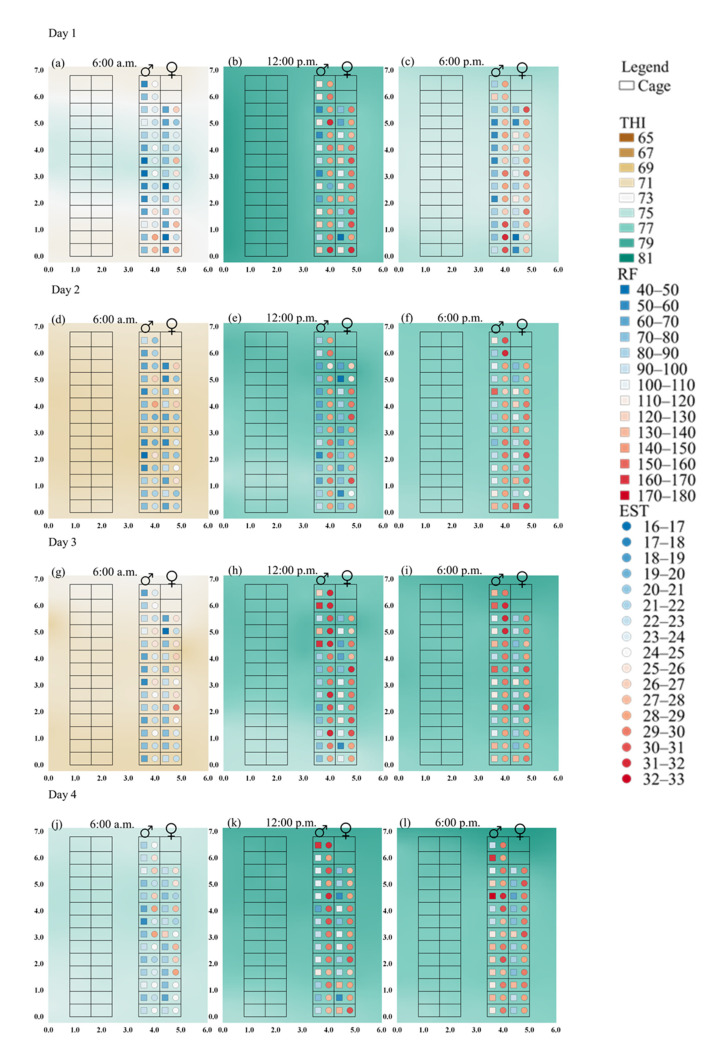

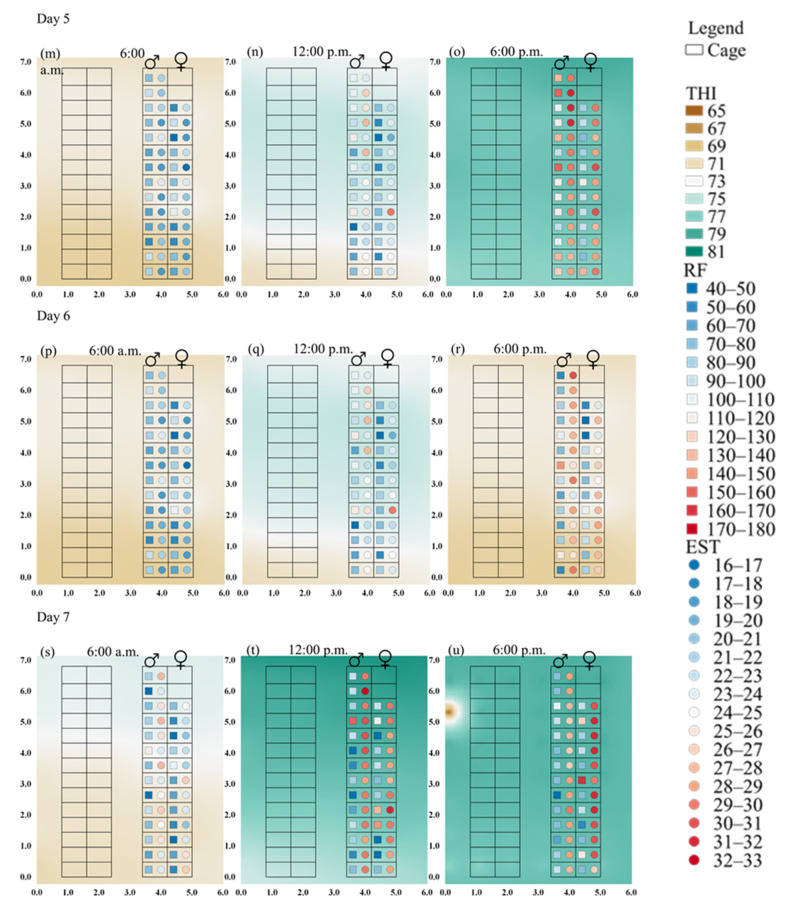
Spatial distribution of the temperature and humidity index (THI), respiratory frequency (RF, breaths.min^−1^), and ear surface temperature (EST, °C) of New Zealand white (NZW) rabbits at 6:00 a.m., 12:00 p.m., and 6:00 p.m. over seven days inside a rabbit house.

The present study analyzed the magnitude and spatial variability of the THI in an NZW rabbit house using values estimated via kriging for the production of isocolor maps with THI spatialization, visualizing the spatial variability of this variable throughout the experimental period inside the facility (Figure 2).

In addition, the RF and ST data were crossed and overlaid onto the final maps of the environmental index (THI) data of the rabbit house, as shown in Figure 2.

According to [39], for efficient production of rabbit meat, the animals must be subjected to a thermoneutral zone (TNZ) of 15 to 25 °C, with an RH of 60–70% [40]. Based on this information, the THI range for the thermal comfort of rabbits was calculated using the equation proposed by [24]. The ideal THI should be maintained between 59 and 74 [9,14] inside an NZW rabbit production facility.

Figure 2 shows that at 12:00 p.m., from the first to the fourth day (Figure 2b,e,h,k) and on the seventh day (Figure 2t), the THI varied between 75 and 81. Therefore, the thermal conditions throughout the rabbit house were above what is considered comfortable for NZW rabbits. However, at 6:00 a.m., the conditions inside the facility were milder, with THI ranging from 65 to 75, showing that, at this time, the animals were possibly thermally comfortable (Figure 2a,d,g,j,m,p,s). At 6:00 p.m., only from the second to the fourth day (Figure 2f,i,l) and on the seventh day (Figure 2u) were the NZW rabbits subjected to thermal conditions outside those recommended as ideal for their rearing.

The environment inside a rabbit house exerts a strong influence on the rabbit production system because it affects the physiological and productive responses of animals. It is crucial to characterize the optimal environmental conditions for rabbits. Investigating the influence of the environment variables on rabbit physiological responses is vital for understanding the effects of thermal conditions on rabbit breeding [11]. The thermal variables inside the rabbit house must be homogeneous in order to potentiate the production performance evaluated through spatialization. In this context, the spatial distribution maps (Figure 2) show that, within the same facility, animals were kept under different environmental conditions due to the variability in THI throughout the rabbit house. This means that, even for the evaluated period (7 days), rabbits were subjected to thermal conditions different from their thermal comfort, which thus harmed the animal’s productive performance. Animals achieve their optimum productivity when they are maintained in a thermoneutral environment, i.e., when the feed energy is not diverted to compensate thermal deviations from the thermoneutral range in order to eliminate or maintain its body temperature [41]. Thus, the environment affected the physiological responses of animals during the evaluated period, and this information can be extrapolated for the entire production cycle.

In a similar experiment, [20] evaluated the magnitude and spatial variability of THI inside a house for laying hens in Antioquia (Colombia). They identified the heterogeneity of the variable’s spatial distribution that was studied in the poultry house. The authors concluded that, through the use of geostatistics, the use of natural ventilation alone was not efficient enough to keep animals under thermal comfort. As such, they suggested that, besides the use of natural ventilation, secondary modifications, such as forced lateral ventilation or mechanics ventilation, should be used to improve the thermal environment and productivity in poultry during the hottest months of the year. This study corroborated the results of the present study, considering that the interaction between THI inside the rabbit house was also a variable that influenced the thermal comfort of rabbits. Therefore, it was possible through the application of geostatistics to observe the areas with better or worse environmental conditions within an NZW rabbit production facility. These problems were precisely corrected with the use of this visual tool in order to improve the thermal comfort of the animals within the rabbit farming system.

Thus, based on studies by [15,20], the use of this method reflects the effect of the thermal environment on the welfare of animals and, therefore, the use of spatial distribution maps is recommended to support producer decision-making to promote improvements in animals house systems.

Rabbits are homeothermic animals and are more sensitive to high air temperature conditions because they are less efficient at thermoregulation by sweating [42], limiting their ability to dissipate excess body heat. Therefore, when rabbits are outside the homeothermy zone, increased RF is one of the necessary mechanisms to stimulate evaporative heat loss [11,43]. Therefore, [23,44] reported that, for rabbits under homeothermy, their RF should vary between 32 and 60 breaths.min^−1^.

Another important parameter to be measured and considered for the assessment of possible stress conditions in rabbits is EST. This is due to the vasoconstriction process that occurs in this part of the body [11,45].

According to the literature, the rabbit ear plays an important role in regulating the temperature because it is the primary cutaneous vascular bed [46].

The EST control can occur as follows: if t_db_ is below 10 °C, the animal will curl up to minimize the heat loss in the total area and decrease EST. However, if the air temperature is high (above 25–30 °C), the animal will stretch to lose as much heat as possible by radiation and convection, as well as increase the EST [47]. Moreover, [48] mentioned in their research that in thermoneutral conditions the EST can vary from 26.4 to 26.9 °C.

At 6:00 a.m. (Figure 2a,d,g,j,m,p,s), it was observed that the RF and EST for most of the NZW rabbits was considered ideal or close to ideal, ranging from 40 to 70 breaths.min^−1^ and 16 at 25 °C, respectively. At 12:00 p.m. (Figure 2b,e,h,k,t) and 6:00 p.m. (Figure 2c,f,i,l,r,u), a large part of the squad had varying RF between 80 to 180 breaths.min^−1^ and EST varying between 26 and 29 ° C, demonstrating that, at these times, NZW rabbits were possibly experiencing thermal discomfort. However, despite the thermal condition of the facility being milder at 6:00 a.m., some rabbits exhibited the acceleration of the RF (Figure 2a,g,j,m,p,s) and EST (Figure 2a,d,g,j,m,s). These RF accelerations are indicated by the reddish colors in the Figures, while the low values of RF are indicated by the bluish colors.

According to [10], the acceleration of the RF could be explained as a response by the animal to the high heat load from the environment during the day. The authors also stated that an increase in the internal temperature of rabbits caused by inadequate environmental conditions accelerated their respiratory movements in an attempt to dissipate excess heat to recover from homeothermy.

Furthermore, [49] submitted 24 NZW rabbits to four ranges of thermal conditions (treatments, T), where T0: 19–26 °C (ambient temperature), T1: 27–28 °C, T2: 31–32 °C, and T3: 35–36 °C for 30 experimental days. Moreover, they classified the THI values obtained as follows: <27.8 °C = absence of heat stress, 27.8–28.9 °C = moderate heat stress, 28.9–30 °C = severe heat stress, and above 30 °C = very severe thermal stress [50] in order to observe changes in the EST of these animals caused by chronic thermal stress conditions. Thus, the authors observed that severe and very severe stress caused changes in the ear temperature of NZW rabbits with a temperature increase of 34 to 34.42 °C compared to the control group (T0) with a temperature of 32.50 °C. This agreed with the obtained results in which animals with exposure to t_db_ of 27.84 to 33 °C at 12:00 p.m. had their ESTs between 26 to 29 °C.

Based on the measured physiological parameters (RF and EST) in this study, both variables showed a direct relationship with the spatial distribution of THI. This is due to their great spatial variability along the rabbit house (Figure 2), demonstrating that the increase of THI led to an acceleration of respiratory movements and to the elevation of the surface temperature of the rabbit’s ear. The latter was an attempt to dissipate the excess heat acquired from the environment.

The interaction between the conditions of the thermal environment of a rabbits’ housing influences an animal’s physiological response. Furthermore, the spatial distribution maps of the THI overlaid with the RF and EST data can be used in practical situations, providing a quick evaluation of the housing conditions and allowing observation of the physiological changes caused in the NZW rabbits due to intensive rearing, as well as poorly designed facilities and inadequate management of the animals.

According to [11,49], when rabbits are raised under stress, eating disorders, weight loss, worsening of feed conversion, changes in water consumption, changes in blood parameters, changes in enzyme profile and hormonal reactions, and imbalance in intestinal health may occur, reducing the digestibility of proteins, energy, and minerals.

Thus, to minimize the harmful effects of environmental stress, one must consider the cage stocking rate, adequate equipment, ventilation, drinking water temperature, and balanced diets, in addition to correct daily management [11,42], to reduce the costs of medications and animal losses.

In this way, the use of the spatial distribution maps climatic indices, such as the temperature and humidity index (THI), makes it possible to provide more detailed information to the producer of changes in physiological responses during the housing, thus allowing for better efficiency in controlling the air conditioning and adjustments in the daily management of the rabbit house in hot weather conditions.

## 4. Conclusions

The use of the geostatistical technique allowed us to verify the occurrence of dependence on the evaluated attributes. They were predominantly strong for the THI thermal environment variable on the days and times analyzed.

The spatial distribution maps of THI allowed us to observe the existence of heterogeneity of the spatial distribution of this parameters in the rabbit houses throughout the evaluated times and days. It was found to form areas where NZW rabbits were exposed to uncomfortable conditions for their development.

The relationship between THI, RF, and EST was evident. The advanced maps showed an increase in THI, which led to an acceleration of respiratory movements and an increase in the surface temperature of the rabbit’s ear. This was an attempt to dissipate the excess calories acquired from the environment.

With RF, EST, and environmental data maps, the critical points of these variables in the rabbit house may be identified clearly and quickly, providing useful information to assist producers in improving the production environment.

## Figures and Tables

**Table 1:** Restricted maximum likelihood method, wave model, and estimated parameters of the experimental semivariograms for the temperature and humidity index (THI) in a New Zealand white (NZW) rabbit house measured over seven days at 6:00 a.m., 12:00 p.m., and 6:00 p.m.

Day	Time	C_0_	C_1_	C_0_ + C_1_	A (m)	DSD		ME	SDME	RE	SDRE
1	6 a.m.	0.018	0.3295	0.348	0.92	95%	Strong	−0.00167	0.1649	−0.00442	1.0004
	12 p.m.	0.198	1.2197	1.418	4.323	86%	Strong	−0.00995	0.5313	−0.00950	1.0446
	6 p.m.	0.000	0.8435	0.844	48.72	100%	Strong	0.00927	0.1629	0.02045	0.7199
2	6 a.m.	0.000	0.3026	0.303	40.08	100%	Strong	0.00516	0.1059	0.01707	0.7337
	12 p.m.	0.094	0.579	0.673	3.60	86%	Strong	0.00276	0.3501	0.00387	1.0102
	6 p.m.	0.015	2.3901	2.405	17.20	99%	Strong	−0.00060	0.1335	−0.00230	1.0216
3	6 a.m.	0.000	0.6141	0.614	13.23	100%	Strong	−0.00008	0.3645	−0.00041	1.0456
	12 p.m.	0.000	2.013	2.013	38.82	100%	Strong	−0.00420	0.3403	−0.00498	0.9370
	6 p.m.	0.000	1.95	1.950	131.42	100%	Strong	−0.00097	0.1645	−0.00211	0.8153
4	6 a.m.	0.009	0.1122	0.121	2.95	93%	Strong	0.00009	0.1113	0.0003	1.0026
	12 p.m.	0.000	4.228	4.228	190.45	100%	Strong	−0.00467	0.2145	−0.00900	0.8701
	6 p.m.	0.000	5.659	5.659	158.34	100%	Strong	0.00286	0.2695	0.00424	0.8386
5	6 a.m.	0.000	0.5387	0.539	42.75	100%	Strong	0.00148	0.1609	0.00365	0.9152
	12 p.m.	0.000	5.604	5.604	333.14	100%	Strong	0.00217	0.1395	0.00454	0.6593
	6 p.m.	0.000	1.086	1.086	116.09	100%	Strong	−0.00061	0.1259	−0.00195	0.8230
6	6 a.m.	0.000	0.889	0.889	21.46	100%	Strong	−0.0058	0.28	−0.0082	1.0000
	12 p.m.	0.000	1.841	1.841	54.88	100%	Strong	−0.01076	0.1993	−0.01696	0.6417
	6 p.m.	0.000	0.889	0.889	21.46	100%	Strong	−0.00580	0.2812	−0.00821	0.8708
7	6 a.m.	0.000	2.105	2.105	136.12	100%	Strong	−0.00135	0.1747	−0.00306	0.8363
	12 p.m.	0.000	12.72	12.720	366.88	100%	Strong	−0.00414	0.2252	−0.00618	0.7147
	6 p.m.	0.000	4.0329	4.033	1.08	100%	Strong	−0.00114	2.0546	−0.00028	1.0203

C_0_—nugget effect; C_1_—contribution; C_0_ + C_1_—threshold; A—reach; DSD—degree of spatial dependence; ME—mean error; SDME—standard deviation of mean error; RE—reduced mean error; SDRE—standard deviation of reduced mean error.

## Data Availability

The data presented in this study are available on request from the corresponding authors.
